# Modulating mitochondrial calcium channels (TRPM2/MCU/NCX) as a therapeutic strategy for neurodegenerative disorders

**DOI:** 10.3389/fnins.2023.1202167

**Published:** 2023-10-20

**Authors:** Gretchen A. Johnson, Raghu R. Krishnamoorthy, Dorota L. Stankowska

**Affiliations:** ^1^North Texas Eye Research Institute, University of North Texas Health Science Center, Fort Worth, TX, United States; ^2^Department of Microbiology, Immunology and Genetics, School of Biomedical Sciences, University of North Texas Health Science Center, Fort Worth, TX, United States; ^3^Department of Pharmacology and Neuroscience, School of Biomedical Sciences, University of North Texas Health Science Center, Fort Worth, TX, United States

**Keywords:** neurodegeneration, mitochondria, aging, ROS, calcium

## Abstract

Efficient cellular communication is essential for the brain to regulate diverse functions like muscle contractions, memory formation and recall, decision-making, and task execution. This communication is facilitated by rapid signaling through electrical and chemical messengers, including voltage-gated ion channels and neurotransmitters. These messengers elicit broad responses by propagating action potentials and mediating synaptic transmission. Calcium influx and efflux are essential for releasing neurotransmitters and regulating synaptic transmission. Mitochondria, which are involved in oxidative phosphorylation, and the energy generation process, also interact with the endoplasmic reticulum to store and regulate cytoplasmic calcium levels. The number, morphology, and distribution of mitochondria in different cell types vary based on energy demands. Mitochondrial damage can cause excess reactive oxygen species (ROS) generation. Mitophagy is a selective process that targets and degrades damaged mitochondria *via* autophagosome-lysosome fusion. Defects in mitophagy can lead to a buildup of ROS and cell death. Numerous studies have attempted to characterize the relationship between mitochondrial dysfunction and calcium dysregulation in neurodegenerative diseases such as Alzheimer’s Disease, Parkinson’s Disease, Huntington’s Disease, Amyotrophic lateral sclerosis, spinocerebellar ataxia, and aging. Interventional strategies to reduce mitochondrial damage and accumulation could serve as a therapeutic target, but further research is needed to unravel this potential. This review offers an overview of calcium signaling related to mitochondria in various neuronal cells. It critically examines recent findings, exploring the potential roles that mitochondrial dysfunction might play in multiple neurodegenerative diseases and aging. Furthermore, the review identifies existing gaps in knowledge to guide the direction of future research.

## 1. Introduction

The small, double-membraned organelle called mitochondria are well-known for being the “powerhouse” of cellular function since they play the vital role of ATP generation and are also important for Ca^2+^ storage and homeostasis, initiation of apoptosis, synthesizing cholesterol, and regulation of mitophagy. ATP and ROS production are linked during oxidative phosphorylation through the electron transport chain (ETC) complexes I and III and increases with high inner mitochondrial membrane potential and high NADH/NAD^+^ ratios (Ramzan et al., 2020). ROS, mainly hydrogen peroxide and superoxide radicals (•O_2_^−^), result from electron leak from the ETC and partial reduction of molecular oxygen, which can damage the cell and are typically resolved through anti-oxidative enzymes. Mitochondria interact with the endoplasmic reticulum, considered the primary calcium storage site, at specific locations called mitochondria-associated membranes (MAMs). To facilitate this interaction, protein tethers keep the membranes at the optimal distance to coordinate shared functions, including phospholipid synthesis, exchange and calcium signaling (Muller et al., 2018; Barazzuol et al., 2021). When dealing with second messengers and mediators affecting cell death/proliferation as well as synaptic transmission, organelle quality control is crucial. One of the key quality control mechanisms is mitophagy, a process of selectively degrading and recycling damaged or excess mitochondria within cells through carefully orchestrated sequential steps. The mitochondria have several pathways for regulation and tagging dysfunctional mitochondria for degradation, namely PTEN-induced putative kinase 1 (PINK1)/Parkin. In a healthy mitochondria, with an intact transmembrane potential, PINK1 gets transported to the inner mitochondrial membrane where it is cleaved by protease and subsequently degraded in the proteasome. Loss of mitochondrial membrane potential triggers PINK1 to stabilize at the outer mitochondrial membrane (OMM) and block protein translocation. Parkin is subsequently recruited, and ubiquitination of OMM proteins occurs for recognition and binding by autophagy receptors (Youle and Narendra, 2011; Sedlackova and Korolchuk, 2019). Mitophagy and mitochondrial biogenesis work simultaneously and in concert to balance the total mitochondria copy number per the needs of the cell. If there is too much biogenesis, there can be an accumulation of ROS, thereby producing damaged mitochondria, which trigger cell death; alternatively, if there is too much mitophagy, there can be overstressing of remaining mitochondria and mitophagic cell death (Sedlackova and Korolchuk, 2019; Cabral-Costa and Kowaltowski, 2020; Cheng et al., 2020).

To add complication, ROS overload often correlates with calcium overload in mitochondria to further exacerbate cell death. Calcium signaling can have different purposes in different tissues, e.g., gene transcription, cell growth, muscle contraction, and egg fertilization, among others. In neurons, calcium signaling functions mainly involve cell differentiation and migration, synaptic transmission and plasticity, vesicle release, cell death and survival, and neuronal-glial communication (Zündorf and Reiser, 2011; Brini et al., 2014; Esteras and Abramov, 2020). Calcium ions [Ca^2+^] are classified as second messengers because they transmit external signals to intracellular targets *via* changes in their cytosolic concentration, which could be either spikes or oscillations. These differences affect the specific downstream effects, altering the amplitudes, frequencies, and spatial locations of calcium ion [Ca^2+^] fluctuations. Deficiencies in calcium signaling perturb synaptic transmission, but overload can be cytotoxic (Zündorf and Reiser, 2011). The resting concentration of calcium in the cytoplasm is typically about 100 nM, and the extracellular concentration is higher in the millimolar range to create a considerable concentration gradient.

Voltage-dependent anion channel (VDAC), also known as mitochondrial porin, controls the passage of metabolites and ions between mitochondria and the rest of the cell and thus mediates metabolic and energetic functions as well as cell survival and death signaling (Shoshan-Barmatz et al., 2010). Both the decrease and increase of VDAC expression have been shown to be detrimental to cellular function as silencing decreased ATP and slowed cellular growth (Abu-Hamad et al., 2006), and overexpression led to apoptosis (Godbole et al., 2003; Zaid et al., 2005). Calcium transport across the OMM is mediated by VDACs and more so in their closed state than open – consistent with VDAC closing being pro-apoptotic (Tan and Colombini, 2007). When there is [Ca^2+^] overload, there is an opening of the mitochondrial permeability transition pore (mPTP), a non-selective channel that spans both inner and outer mitochondrial membranes. The problematic channel has been implicated as a mechanism of cell death and has since become a target of interest. Several different types of calcium channels are found in the plasma, ER, and mitochondria membranes that respond to various signals. The two types of calcium channels primarily utilized by the mitochondria for [Ca^2+^] homeostasis are mitochondria calcium uniporters (MCU) and Na^+^/Ca^2+^ exchangers (NCX) (Contreras and Satrustegui, 2009; Muller et al., 2018; Tong et al., 2018; Semyanov, 2019; Esteras and Abramov, 2020; Ruiz et al., 2020).

Neurons, being high-energy-demanding cells, naturally possess a greater number of mitochondria. Consequently, they are more susceptible to dysfunctions related to mitochondrial activities. For example, diseases with mutations in the mitochondrial genome alter overall mitochondrial function and often present symptoms in the nervous system (sensorineural loss, stroke, ataxia, parkinsonism, optic atrophy, migraine, dementia) (Mandemakers et al., 2007; Gorman et al., 2016). A compromise in mitochondrial function can impact calcium channels within the mitochondria, thereby disrupting calcium signaling. When mitochondria are dysfunctional and calcium homeostasis is dysregulated, it can lead to excitotoxic, calcium-mediated cell death. Characterizing neurodegeneration by disease has been difficult due to the multifactorial nature of neurodegeneration – it is the later stages that are noticed due to emerging symptoms; however, the specific cellular mechanisms at this point in the disease progression are often similar. The various contributors to cell death are of interest to try to arrest the disease before irreversible damage is done. A major contributor in the context of many neurodegenerative disorders is mitochondrial dysfunction, and another less-discussed theory is calcium dysregulation. As underscored above, these two functions are interrelated and are likely to influence one another. It is, therefore, reasonable to conceive ways to overcome dysregulated mitochondrial function by the modulation of channels that regulate calcium homeostasis. This could open the door to a new branch of therapeutics targeting different neurodegeneration types that currently lack effective treatments.

### 1.1. Mitochondrial calcium uniporter

To reduce the concentration of cytoplasmic [Ca^2+^], Mitochondrial Calcium Uniporters (MCUs), located in the Inner Mitochondrial Membrane (IMM), uptake calcium. This calcium is transported into the mitochondrial matrix by ion channels for storage. The MCU has a wide range of capacity; hence, there must be a high enough concentration before significant transport can occur. This could indicate a safety mechanism to prevent overload of [Ca^2+^]in the cytosol; however, beyond a certain concentration threshold, the elevation of mitochondrial [Ca^2+^] might be damaging to the mitochondria. The molecular composition of the uniporter has recently been parsed out to reveal a protein complex containing a pore-forming component and several regulatory units (MICU1 & MICU2), the expression of which may vary slightly based on tissue type. Reports have shown that an MCU enhancer, MICU3, is highly expressed in the brain and dimerizes with MICU1 through disulfide bond formation (Patron et al., 2019; Wang et al., 2023). Numerous studies indicate that upregulation of MICU3 enhances the uptake of mitochondrial [Ca^2+^] (Granatiero et al., 2019; Cabral-Costa and Kowaltowski, 2020; Esteras and Abramov, 2020), while its downregulation results in decreased uptake and accumulation of ROS (Wang et al., 2023). This makes MICU3 a compelling candidate for therapeutic potential.

### 1.2. Na^+^/Ca^2+^ exchanger

The Na^+^/Ca^2+^ Exchanger (NCX) is a low affinity, high-capacity sodium-calcium exchanger and transmembrane protein critical in regulating calcium ions [Ca^2+^] concentration within cells. To mediate mitochondrial [Ca^2+^] efflux, the NCX found in the IMM employs the electrochemical gradient of Na^+^ to exchange three Na^+^ ions for one [Ca^2+^] ion. The general efflux rate is slower than MCU’s influx rate, indicating a significant role for NCX in mitochondrial [Ca^2+^] homeostasis. These exchangers are also reversible and can indirectly interact with calpain-induced degradation, pH, and protein kinases C and A. Kostic and Sekler (2019) delved into the functional properties and mode of regulation of mitochondrial NCX and emphasized the need for a more specific/selective blocker to study the physiological role of NCX in different cell types (Kostic and Sekler, 2019). Despite the limited understanding of the subject, significant therapeutic potential still makes it an area of interest. Knockout models have demonstrated their importance through the lethality of deletion of NCX in myocardial tissue and protection against damage when overexpressed (De Oliveira et al., 2019; Kostic and Sekler, 2019; Esteras and Abramov, 2020). The ability to utilize sodium to reduce the calcium load in mitochondria could be useful in preventing or slowing neurodegeneration.

## 2. Neurodegenerative diseases and aging

Numerous neurodegenerative diseases have been investigated, considering mitochondrial dysfunction and calcium dysregulation. In this review, the focus is on the critical findings in Alzheimer’s Disease (AD), followed by a brief discourse about similar findings in Parkinson’s Disease (PD), Huntington’s Disease (HD), and spinocerebellar ataxia (SCA) research.

AD is commonly characterized as the formation of amyloid plaques (abnormally high deposition of amyloid beta peptides) and neurofibrillary tangles resulting from hyperphosphorylation of Tau, a microtubule-associated protein. The exact mechanisms remain unclear partly due to recent findings that disruption of [Ca^2+^] homeostasis can precede amyloid plaques and neurofibrillary tangles (Huang et al., 2022). There have also been findings suggesting misfolded proteins (including amyloid-β and Tau) alter [Ca^2+^] homeostasis, indicating a wide range of causes. Multiple sources found correlations between NCX loss and [Ca^2+^] dysregulation with AD progression and proposed rescue through NCX expression or inhibition of MCU by blocking mPTP opening (Tong et al., 2018; Cabral-Costa and Kowaltowski, 2020; Calvo-Rodriguez et al., 2020; Esteras and Abramov, 2020).

PD is characterized by the progressive loss of dopaminergic neurons in the substantia nigra, which manifests as deficits in both motor and non-motor functions. The primary source of HD is attributed to a repeat expansion of CAG trinucleotides in the first exon encoding the huntingtin protein, resulting in a mutant protein with numerous repeats, leading to neuronal loss along with disruptions in motor and cognitive function. The similarity in AD, PD, and HD is the presentation of neuronal death following mitochondrial [Ca^2+^] alteration and oxidative imbalance; the differences lie in the component(s) of maintenance of mitochondrial bioenergetics and function that is impaired (Sheehan et al., 1997; Sousa et al., 2003; Hariharan et al., 2014; Rodriguez et al., 2022). A link between impairment in complex I activity and neurodegeneration was identified in PD patients (Sheehan et al., 1997). Additionally, inhibiting complex I in neurons of lab animals led to a rapid depolarization, followed by oxidative and [Ca^2+^] imbalance (Sousa et al., 2003). Those with HD have an altered huntingtin protein essential in mitochondrial bioenergetics maintenance. Without the properly functioning huntingtin protein, there is a reduction in electron transport activity, exhibited in animal models with inhibited complex II activity (Hariharan et al., 2014).

Spinocerebellar ataxia (SCA), a heterogeneous group of progressive neurodegenerative diseases of the cerebellum, has long had implications for dysregulated calcium homeostasis (Liu et al., 2009; Sullivan et al., 2019). Some types have newly described mutations in genes, such as voltage-gated calcium channel subunit alpha 1 G (CACNA1G) and glutamate metabotropic receptor 1 (GRM1) (Watson et al., 2017).

The preceding examples of neurodegeneration mechanisms demonstrate how impairments in different components relating to mitochondrial bioenergetics and calcium homeostasis (stemming from either disease or aging) can present similar damage-inducing malfunctions that cascade into neurodegeneration. During aging, over time and in the absence of disease, cells progressively become senescent – i.e., they either lose optimal function and/or have damaged or impacted cellular processes but have not yet begun to die. Many individual alterations can make up cellular senescence or aging, but some that occur are directly related to the mitochondria and calcium signaling. One alteration found is the downregulation of the mitochondrial calcium uptake family member 3 (MICU3) in skeletal muscle of aged mice, which was associated with increased oxidated stress and apoptosis and the reconstitution of MICU3 enhanced antioxidants, decreased apoptosis, and prevented mitochondrial ROS accumulation (Yang et al., 2021).

Since different cell types have differentially expressed genes and proteins, generally conserved processes may vary to fit the needs of the cell, such as mitochondria distribution and activity. Mitochondria have been shown to move to elevated [Ca^2+^] sites and regions of high ATP demand to provide buffering capacity and supply energy. Until recently, astrocyte processes were thought to be too small to accommodate mitochondria because of the small size of the astrocyte, but newer studies indicated otherwise (Jackson and Robinson, 2018). Complex I assembly into the (I/III) supercomplex is reduced in astrocytes compared to neurons, resulting in lower complex I activity and respective downstream effects connected to astrocyte calcium dysfunction reports in AD, PD, and HD (McAvoy and Kawamata, 2019; Okubo, 2020; Okubo and Iino, 2020).

Another aspect worth considering is the comparison between the Peripheral Nervous System (PNS) and the Central Nervous System (CNS), given their differences in axonal regeneration capacity (Smith et al., 2020). Fecher et al. (2019) demonstrated that cell-type-specific regulation could occur even in the most basic mitochondrial functions in the CNS. They speculated and suggested that this mitochondria diversity imbues properties that contribute to the unique function of different brain cell types and selective vulnerability during disease (Fecher et al., 2019). In the context of SCA, cerebellar Purkinje cells (GABAergic inhibitory neurons) are most affected by disturbed calcium homeostasis and mitochondrial dysfunction (Kreiner et al., 2010; Egorova et al., 2015, 2023). These could be an example of selective vulnerability where large amounts of efficient mitochondria are required.

Lastly, calcium and mitochondria dysregulation have been implicated in various optic neuropathies (Mueller et al., 2011). In animal models of glaucoma, an accumulation of mitochondria was observed in the optic nerve (ganglion cell axons), specifically in the prelaminar and laminar regions, which was thought to result from either mechanical compression or axoplasmic stasis (Bristow et al., 2002). Rather, it has been suggested that mitochondrial activities are different across tissues and in specific regions to compensate for and maintain function; thus, the unmyelinated optic nerve may be susceptible to mitochondrial dysfunction because of higher energy demand (Bristow et al., 2002; Yu Wai Man et al., 2005; Maresca et al., 2013). Additionally, the calcium-sensitive apoptotic proteins Calcineurin and Calpain have been implicated not only in glaucoma (Huang et al., 2010) but also in other neurodegenerative diseases, contributing to calcium dysregulation (Mueller et al., 2011). Inhibiting these proteins has been shown to prevent the late-stage activation of apoptosis (Loetscher et al., 2001; Wu et al., 2004). However, employing calcium channel blockers as a therapeutic strategy could potentially be more effective by addressing dysregulation at an earlier stage.

## 3. Recent studies and potential therapeutic targets

**Figure 1:**
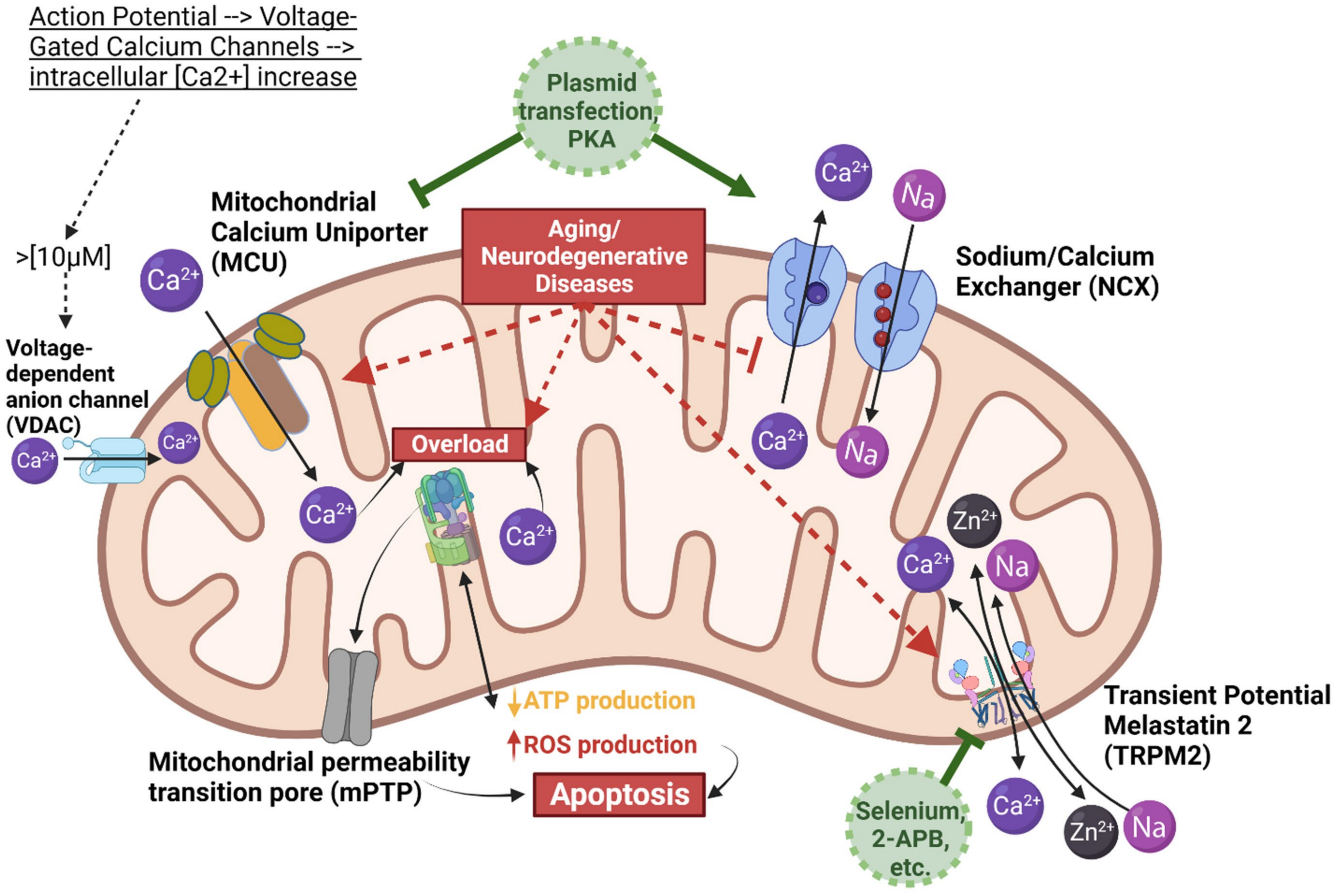
Mitochondrial calcium homeostasis. This scheme illustrates the mitochondrion and the key components involved in calcium homeostasis, demonstrating the balance between calcium influx *via* the mitochondrial calcium uniporter (MCU) and efflux *via* the Sodium Calcium Exchanger (NCX). The MCU is depicted in tan/brown, calcium in dark purple, NCX in blue, and sodium flow in light purple. The grey arrows do not represent direct actions but rather illustrate the downstream effect of calcium on reactive oxygen species (ROS) production and the opening of the mitochondrial permeability transition pore (mPTP). When cytosolic calcium concentration exceeds 10 micromolar, voltage-dependent anion channels (VDACs) transport calcium into the intermembrane space. Subsequently, MCU, NCX, and TRPM2 mediate ion movements between the intermembrane space and the mitochondrial matrix. Given the higher rate of calcium influx compared to efflux, calcium ions can easily accumulate, leading to ATPase overload, excess ROS production, and the opening of mPTP, which can trigger apoptosis. To prevent calcium overload, two potential interventions are proposed: using a blocker to reduce the rate of calcium influx, or employing protein kinase A (PKA) or plasmid transfection to enhance NCX expression and thus increase the rate of calcium efflux. Created in BioRender.

Characterizing the mitochondrial mechanisms that contribute to neurodegeneration is sorely needed to aid in the identification of potential therapeutic targets. Relieving mitochondrial calcium dysregulation has been approached from multiple angles, such as upregulating calcium-binding proteins Calretinin and Calbindin-D28K to buffer free calcium (La Barbera et al., 2022) as well as targeting pathways such as mTORC1-SKN-1-Nrf (Ryan et al., 2022). Some of the most promising targets for mitoprotection (Figure 1) include NCX and MCU, as well as a channel that has only been found in neuronal mitochondria to aid with zinc (Zn^2+^) uptake: transient potential melastatin 2 (TRPM2). This channel is also capable of [Ca^2+^] uptake and has gained prominence in AD studies. However, there is a notable prevalence of reviews over empirical findings (Jiang et al., 2018; Ataizi et al., 2019). A review by Jiang et al. discusses recent theories involving TRPM2 being used in neurons to decrease intracellular zinc uptake into the mitochondria, which can lead to neuronal degeneration. The authors suggest more evidence is needed to determine if the channel mediates Zn^2+^ flux from lysosomes or into mitochondria (Jiang et al., 2018). Next, a few studies investigating NCX, MCU, or TRPM2 as therapeutic targets are discussed.

Several key findings on different aspects of mitochondrial [Ca^2+^] homeostasis raise the potential for identifying therapeutic targets to protect against neurodegeneration. In 2019, a group investigated the neuroprotective effects of melatonin and selenium against docetaxel damage to the brain and hippocampus. Docetaxel is meant to treat glioblastoma (an aggressive cancer of the brain that forms in astrocytes) but has detrimental effects on the brain compared to other tissues (Ataizi et al., 2019). The drug was found to induce excessive ROS production and activate caspase −3 and − 9 to promote apoptosis, potentially through cytosolic calcium overload. Melatonin and selenium were shown to stimulate antioxidant response and inhibit TRPM2, a calcium-permeable, non-selective cation channel, by facilitating the neutralization of ROS into less harmful products. The fluorescence data suggested a more significant improvement mediated by selenium than melatonin. Intriguingly, when treating hippocampal cells with 2-APB, a known TRPM2 channel blocker, there was a decrease in fluorescence, indicating decreased [Ca^2+^], suggesting a role in calcium homeostasis and supporting the potential of TRPM2 being in the mitochondria and/or involved with mitochondria in neurons. Another study found that amyloid-β inducing ROS can activate TRPM2 to alter intracellular Ca^2+^/Zn^2+^ homeostasis (Jiang et al., 2018). Selenium has since been used in microglia to mitigate interferon-gamma’s activation of TRPM2 and, when combined with 2-APB, showed potentiated effects of TRPM2 inhibition and decreased calcium influx (Akyuva et al., 2021). Carvacrol has also been recently proposed as an effective TRPM2 antagonist in SH-SY5Y neuronal, BV-2 microglial, and HEK293 (human epithelial kidney derived) cells (Nazıroğlu, 2022). In the context of the eye, specifically retinal pigment epithelial cells, Selenium reduced TRPM2 activity following hypoxia (Özkaya et al., 2021), and Carvacrol attenuated TRPM2 activity following high glucose insult (diabetes mellitus model) (Daldal and Nazıroğlu, 2022). Considering these findings, there is an implication that multiple stimuli (such as amyloid-β, docetaxel, INF-gamma, hypoxia, or high glucose) generate ROS as a second messenger to produce TRPM2 dysfunction. This highlights the dynamic nature of regulation and the need to characterize further specific mechanisms involving TRPM2.

Several studies have investigated the modulation of NCX in neurons and have demonstrated the rescue of mitochondrial function in models of AD-associated pathology (Jadiya et al., 2019), PD-associated pathology (Sisalli et al., 2022), and cerebral ischemia (Abedinzade et al., 2022). When NCX was knocked out of hippocampal cells, the positive effects of PDE2 inhibitors (which rescue calcium efflux by diminishing mitochondrial cAMP) were diminished, confirming a relationship between NCX and PDE2-dependent neuronal survival (Rozenfeld et al., 2022). In human SH-SY5Y cells (neuronal-like blastoma), an increase of NCX expression and activity that corresponded with improved mitochondrial functions was observed following rotenone and 6-hydroxydopamine treatment (Abedinzade et al., 2022). Another connection to mitochondrial calcium homeostasis is PD-associated LRRK2 deficiency having calcium efflux rescued by NCX upregulation (Ludtmann et al., 2019). These studies highlight a link between NCX expression/activity and calcium homeostasis and that it has a noticeable effect on cellular function.

One group performed immunoblot analyses of proteins associated with mitochondrial calcium exchange in brain samples from diagnosed AD patients and found decreased expression of NCX and remodeling of MCU components. Next, the group used an animal model with NCX knockout and found compelling data indicating accelerated AD pathology. A model with AD mutations was utilized to rescue from the AD pathology by increasing neuronal NCX mRNA and protein expression at 4 weeks of age. The results were impressive – genetic rescue *via* introducing a vector containing the transcript for NCX entirely removed age-associated cognitive decline and reduced the expected neuronal pathology. Mice overexpressing NCX tested similarly to healthy controls, suggesting that this novel treatment for neurodegeneration is sufficient for suppressing a cognitive decline in even advanced stages. Mitochondrial function was investigated in response to the restoration of calcium efflux and was found to be improved. In the discussion, the authors explored the downregulation of calcium efflux in early AD pathogenesis, positing it as a possible compensatory mechanism to meet the increased demand for dehydrogenase activity and ATP production. They suggested that as the need for metabolic signaling grew, calcium efflux decreased while calcium uptake increased to supplement [Ca^2+^] signaling (Jadiya et al., 2019).

Some groups have investigated the modulation of MCU in Parkinson’s and Alzheimer’s models. A 2022 publication found that patients with PD had variants in Miro1, a Rho GTPase that aids calcium buffering and mitophagy. The inhibition of MCU resulted in features characteristic of the mutated Miro1 genotype calcium response, demonstrating Miro1’s ability to modulate calcium *via* the MCU (Schwarz et al., 2022). Another study found that MCU knockdown in hippocampal neurons improved memory, decreased neuroinflammatory responses, and improved PINK1-Parkin signaling (Cai et al., 2022). Alternatively, a group that knocked down MCU in excitatory neurons found disruption in neuronal network oscillations that require high mitochondrial performance (Kann et al., 2014). A 2022 study on the relationship between action potential firing and MCU calcium uptake in excitatory neurons of the cortex and hippocampus found that the activation of MCU was matched to enhanced firing rate and likely acts through metabolic regulation and excitability control (Groten and MacVicar, 2022). This could mean that MCU modulation would be more beneficial in cell types that do not rely on network oscillations.

Despite the numerous gaps in our understanding of neurodegeneration and the mechanisms involved in various calcium channels and responses to stimuli, a handful of promising treatments still show potential. It would be worth investigating a combination of treatments to combat the dysregulation of mitochondrial calcium, such as targeting TRPM2 or MCU (depending on the disease) for inhibition in addition to increasing NCX expression (Figure 1). It is worth noting that these studies have primarily been in cells and a few *in vivo*, so further research is needed to understand any potential drawbacks of the proposed treatments fully.

## 4. Conclusion

With aging, most cells in the body become more susceptible to mutations, mitochondrial damage, and associated diseases. By virtue of their high metabolic rate and oxygen consumption, neurons are particularly susceptible to mitochondrial injury and degeneration. Not surprisingly, one of the most prominent and devastating effects of age is the degeneration of neurons and subsequent loss of memory and motor function. There are numerous ways to analyze mechanisms contributing to neurodegeneration in various neurodegenerative diseases. One active area of investigation is the involvement of mitochondria in regulating calcium, ROS, zinc, and sodium. Regardless of the inciting cause of degeneration in aging, AD, PD, and HD, it has become clear that a common denominator leading to cell death is the dysregulation of mitochondrial [Ca^2+^] homeostasis, which makes it an ideal therapeutic target. The data reviewed here highlight the potential for developing treatments for neurodegeneration in animal models. The main trigger of dysfunction in the mitochondria from disruption of calcium homeostasis remains to be determined. This could involve mitochondrial calcium uniporter (e.g., MICU3), the sodium-calcium exchanger, the TRPM2 cation channel, and specific mechanisms of TRPM2 alteration and NCX downregulation. Increasing NCX expression and using a TRPM2-specific blocker (e.g., selenium, 2-APB) may be promising approaches for *in vivo* experiments aimed at developing new therapies. More studies need to be done to support these findings and elucidate the side effects of novel candidate drugs.

## Author contributions

GJ was responsible for the concept and writing of the current work. DS and RK contributed to the manuscript by providing guidance, expertise, as well as by correcting and editing the text and refining the scientific content. All authors contributed to the article and approved the submitted version.
